# HMGB1: a double-edged sword and therapeutic target in the female reproductive system

**DOI:** 10.3389/fimmu.2023.1238785

**Published:** 2023-08-18

**Authors:** Yu Ren, Damin Zhu, Xingxing Han, Qiqi Zhang, Beili Chen, Ping Zhou, Zhaolian Wei, Zhiguo Zhang, Yunxia Cao, Huijuan Zou

**Affiliations:** ^1^ Reproductive Medicine Center, Department of Obstetrics and Gynecology, The First Affiliated Hospital of Anhui Medical University, Hefei, China; ^2^ National Health Commission (NHC) Key Laboratory of Study on Abnormal Gametes and Reproductive Tract, Anhui Medical University, Hefei, China; ^3^ Key Laboratory of Population Health Across Life Cycle, Anhui Medical University, Hefei, Anhui, China; ^4^ Anhui Key Laboratory of Reproductive Health and Genetics, Anhui Medical University, Hefei, Anhui, China; ^5^ Biopreservation and Artificial Organs, Anhui Provincial Engineering Research Center, Anhui Medical University, Hefei, Anhui, China

**Keywords:** HMGB1, pregnancy complications, female reproductive system diseases, inflammation, anti-HMGB1

## Abstract

HMGB1 that belongs to the High Mobility Group-box superfamily, is a nonhistone chromatin associated transcription factor. It is present in the nucleus of eukaryotes and can be actively secreted or passively released by kinds of cells. HMGB1 is important for maintaining DNA structure by binding to DNA and histones, protecting it from damage. It also regulates the interaction between histones and DNA, affecting chromatin packaging, and can influence gene expression by promoting nucleosome sliding. And as a DAMP, HMGB1 binding to RAGE and TLRs activates NF-κB, which triggers the expression of downstream genes like IL-18, IL-1β, and TNF-α. HMGB1 is known to be involved in numerous physiological and pathological processes. Recent studies have demonstrated the significance of HMGB1 as DAMPs in the female reproductive system. These findings have shed light on the potential role of HMGB1 in the pathogenesis of diseases in female reproductive system and the possibilities of HMGB1-targeted therapies for treating them. Such therapies can help reduce inflammation and metabolic dysfunction and alleviate the symptoms of reproductive system diseases. Overall, the identification of HMGB1 as a key player in disease of the female reproductive system represents a significant breakthrough in our understanding of these conditions and presents exciting opportunities for the development of novel therapies.

## 1 Introduction

High-mobility group proteins (HMG) were first extracted and identified in the bovine thymus in 1973 and named for their high mobility in gel electrophoresis (1). Based on their functional sequence motif characteristics, HMG proteins are divided into three superfamilies: HMGB, HMGA, and HMGN (2). As the most abundant protein among all HMG family members, HMGB1 is a multifunctional protein that plays a crucial role in various cellular processes (3). As a DNA-binding nuclear factor, it regulates the transcriptional activity of genes, controls DNA replication and repair, and facilitates telomere maintenance and nucleosome assembly (4). In addition to its intracellular functions, HMGB1 can be transferred into the extracellular environment (5). the released or secreted HMGB1 functions as a damage-associated molecular pattern (DAMPs) that can interact with pattern recognition receptors (PRRs), such as receptors for advanced glycation end-products (RAGE) and toll-like receptors (TLRs) (6). Under normal conditions, HMGB1 can promote cell proliferation and differentiation, promote inflammatory reaction and immune response, participate in tissue repair and regeneration, and regulate gene expression and transcription (7–10). In pathological conditions, HMGB1 plays a crucial role in amplifying the inflammatory response and driving the pathogenesis of many diseases. For instance, HMGB1 has been implicated in several malignancies, including breast, lung, and colorectal cancers (11). Overall, these findings highlight the importance of HMGB1 in multiple cellular processes and suggest that it may be a potential therapeutic target for various diseases.

Pregnancy is a complex physiological process. During pregnancy, the maternal immune system is constantly changing in response to fetal development and environmental signals. Inflammation is a critical mechanism in the establishment of pregnancy, the initiation of labor, and the development of many pregnancy complications (12, 13). Normal pregnancy has three distinct immunological phases that are characterized by distinct biological processes. The first trimester of pregnancy is a pro-inflammatory phase. Early pregnancy includes events such as decidualization, implantation, trophoblast development, and placental growth. At this stage, the blastocyst breaks through the uterus lining and invades the endometrial tissue (14). Therefore, an inflammatory environment is necessary for repairing the uterine epithelium and removing cellular debris. In the second trimester of pregnancy, the fetus grows rapidly while the mother, placenta, and fetus work together to induce an anti-inflammatory state. During the final trimester of pregnancy, the baby’s organs fully develop and the mother’s body prepares for birth. The uterus contracts due to a pro-inflammatory environment in this stage, which helps with the expulsion of the baby and placenta (15). Whether before or at any stage of pregnancy, immune disorders can have a significant impact on pregnancy outcomes.

**Figure 1:**
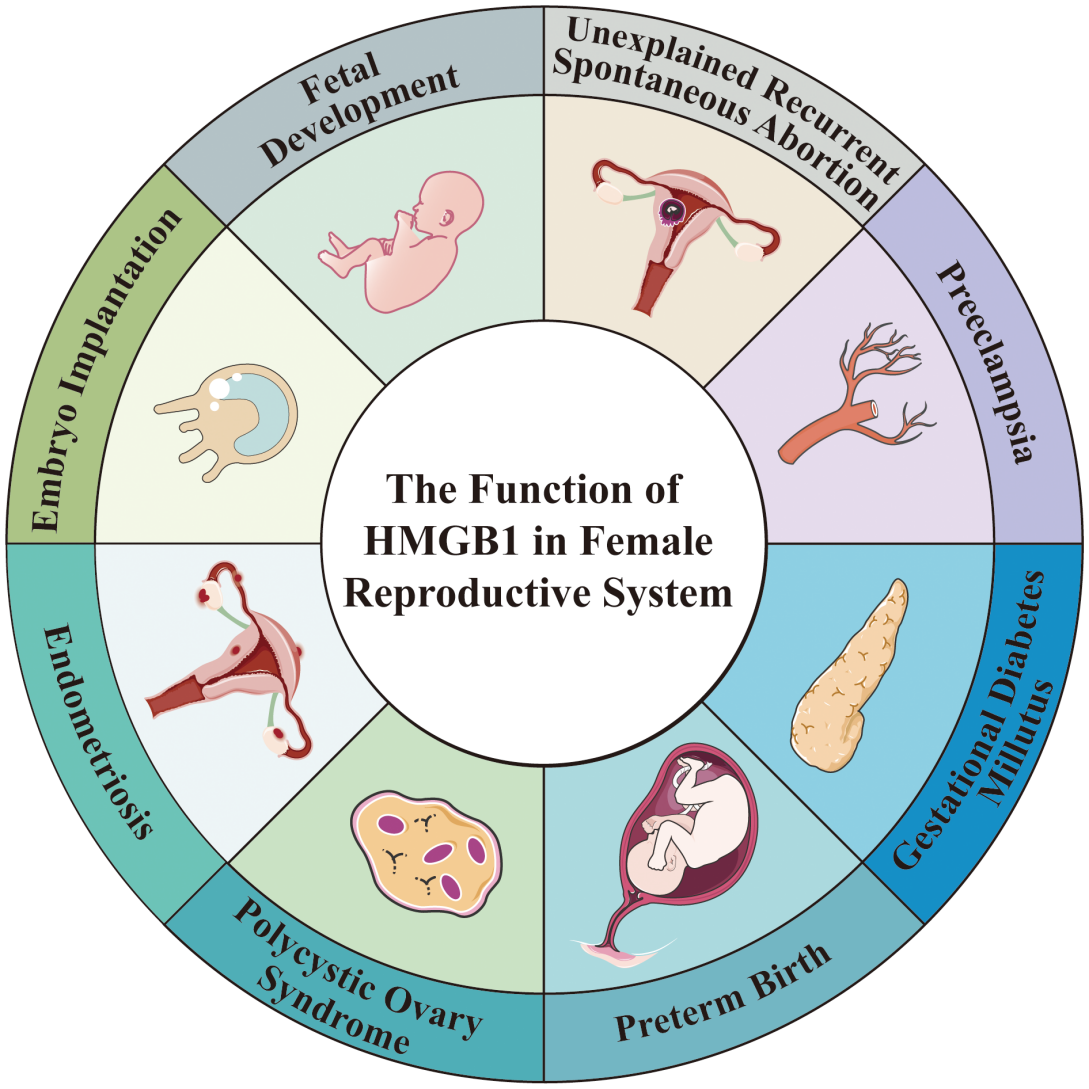
The Function of HMGB1 in Female Reproductive System. HMGB1 is involved in fetal development and embryo implantation, as well as a variety of diseases in female reproductive system, such as preeclampsia, preterm birth, unexplained recurrent spontaneous abortion, gestational diabetes millutus, polycystic ovary syndrome, endometriosis.

Over the last decade, extensive studies have demonstrated that HMGB1 plays a vital role throughout pregnancy and is involved in various diseases of the female reproductive system (**Figure 1**). HMGB1 promotes uterine decidualization and embryo implantation, and embryonic development in the first trimester (16–19), and cervical ripening and delivery in the third trimester (20). However, high level of HMGB1 levels may result in female reproductive disorders, including recurrent spontaneous abortion (RSA) (21), gestational diabetes mellitus (GDM) (22), preterm birth (PTB) (23), preeclampsia (PE) (24), polycystic ovary syndrome (PCOS) (25), and endometriosis (26). Further research is needed to determine the precise role of HMGB1 in normal pregnancy and the development of reproductive disorders. HMGB1 may serve as a valuable biomarker for the early prediction of these diseases and provide new ideas for their prevention and treatment. This review provides an overview of the function of HMGB1 and explores available HMGB1 inhibitors in the female reproductive system.

## 2 The overview of HMGB1 as a DAMP

**Figure 2:**
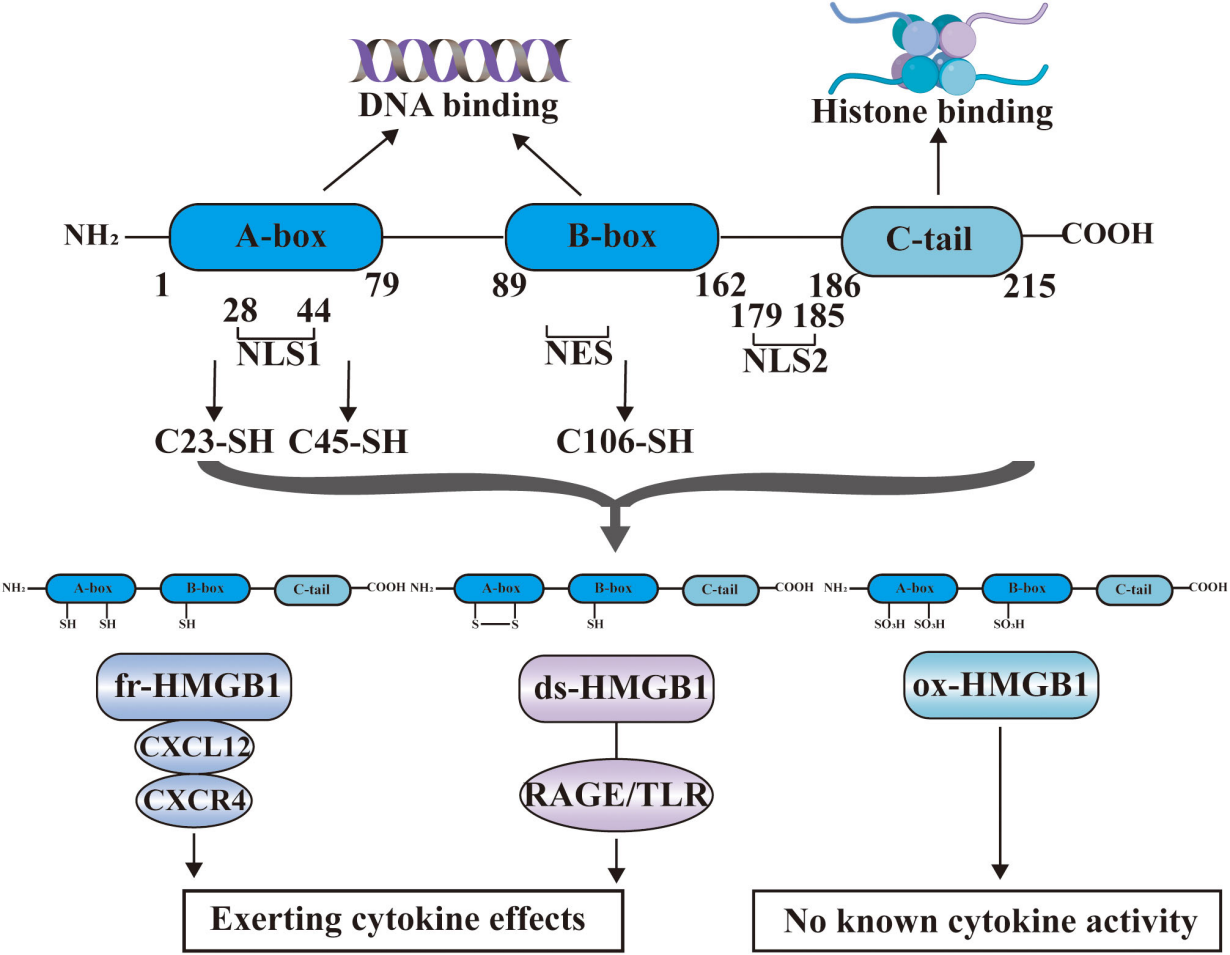
The structure of HMGB1. HMGB1 consists of HMG A box (9-79 amino acids), B box (89-162 amino acids), a highly negatively charged C-terminal tail (186-215 amino acids) and a short N-terminal region. The A and B boxes in the nucleus can bind DNA, and the C-terminal region can bind histones. HMGB1 has two nuclear localization signal regions (NLS1 and NLS2) and one nuclear export signal (NES). HMGB1 has three cysteine residues at amino acids C23, C45, and C106. C23 and C45 are located in A box, while C106 is located in B box. The redox state of these three cysteines determines that HMGB1 has three redox forms: (1) Fully reduced HMGB1 (fr-HMGB1) can bind to CXCL12 and then stimulate leukocyte recruitment through the CXCR4 receptor. (2) Partially reduced HMGB1 called disulfide bond HMGB1 (ds-HMGB1) can trigger inflammatory responses via receptors including RAGE and TLRs. (3) Completely oxidized HMGB1 (ox-HMGB1) has no known cytokine activity.

HMGB1 is an evolutionarily highly conserved nuclear non-histone DNA binding protein that was discovered in 1973. The structure of HMGB1 is shown in **Figure 2**, and consists of two consecutive positively charged DNA binding domains, called HMG A box (9-79 amino acids) and B box (89-162 amino acids), a highly negatively charged C-terminal tail (186–215 amino acids) and a short but functionally significant N-terminal region (27).

In the nucleus, the A and B boxes bind to DNA, whereas the C-terminal region binds to histones. HMGB1 binds to DNA with low affinity. As a shuttle protein translocating between the nucleus and cytoplasm, HMGB1 has two nuclear localization signal regions (NLS1 and NLS2) and one nuclear export signal (NES) (28). HMGB1 is the most mobile protein in the nucleus, and can pass through the nucleus and enter the cytoplasm within 1-2 seconds (3, 29). HMGB1 is transferred into the extracellular environment through two different mechanisms: (1) Active secretion: Immune cells and some other cells like epithelial cells and endothelial cells can actively secrete HMGB1 when they are stimulated by various factors (30–33). These factors include lipopolysaccharide (LPS), pathogen infections, and endogenous host stimuli (34, 35). This active release of HMGB1 can occur through two models. In one model, the cells with stimuli directly secrete HMGB1 into the extracellular space (36). In the other model, HMGB1 is packaged into intracellular vesicles, such as exosome. These vesicles then fuse with the cell membrane, leading to the release of HMGB1 outside the cell (37). (2) Passive release: HMGB1 can also be passively released from various types of cell death in response to different stimuli or damages. These types of cell death include necrosis, necroptosis, apoptosis, autophagy-dependent cell death, NETosis, pyroptosis, PANoptosis and ferroptosis (38–44). Extracellularly, HMGB1 participates in inflammatory response as DAMPs, thereby, playing a role in the occurrence and development of various physiological processes and diseases (45). The B box of HMGB1 is a functional domain that recognizes pattern recognition receptors (PRRs) and can induce macrophages to secrete proinflammatory cytokines (6). In contrast, A box can antagonize the activity of cytokines, and A box alone can act as a competitive antagonist of HMGB1 and inhibit the activity of HMGB1 (46).

HMGB1 contains three cysteine residues at amino acids C23, C45, and C106 (47). C23 and C45 are located in A box, where a disulfide bond can be formed between them, and C106 is located in B box (48). The redox state of these three cysteines determines whether HMGB1 has three redox forms that can regulate the extracellular activity of HMGB1 (47). Fully reduced HMGB1 (fr-HMGB1) has three conserved cysteines containing thiol groups that can form a complex with other chemokines, such as CXCL12 and stimulate leukocyte recruitment through the CXCR4 receptor (49). Partially reduced HMGB1 called disulfide bond HMGB1 (ds-HMGB1), has a disulfide bond between C23 and C45, which can trigger inflammatory responses via TLR2/4 or other receptors such as RAGE and TLR9 (50, 51). Completely oxidized HMGB1 (ox-HMGB1) has no immunological competence because it cannot activate macrophages or dendritic cells (52–54). In addition, HMGB1 is also subject to various types of post-translational modifications (PTMs), including acetylation, methylation, phosphorylation, N-glycosylation, phosphorylation, and ADP-ribosylation, which determine their subcellular localization and biological activity (27).

HMGB1 released outside cells binds to cell surface receptors on target cells as a DAMP (6). To date, a large number of studies have reported the related receptors of HMGB1, including toll-like receptors (TLR) 2-4/7/9, the receptor for advanced glycation end products (RAGE), integrin, cluster of differentiation 24 (CD24), T-cell immunoglobulin and mucin-3 (TIM3) and C-X-C chemokine receptor type 4 (CXCR4) (49, 55–57). So far, TLR4 and RAGE have been extensively studied and proven to be specific HMGB1 receptors in a large number of studies.HMGB1 combined with RAGE or TLR4 can activate a variety of signaling pathways, including the mitogen-activated protein kinase (MAPK) signaling pathway to mediate the occurrence of immune reactions such as autophagy (58), pyroptosis (59) and apoptosis (60) or the amplification of inflammation, thus playing a crucial role in diabetes, epilepsy, tumor and other diseases (3). Similarly, HMGB1 also plays a role in normal pregnancy and disease in female reproductive system by interacting with its receptors.

## 3 The role of HMGB1 in normal pregnancy

### 3.1 HMGB1 facilitates embryo implantation

Embryo implantation occurs when a fertilized egg attaches to the uterine lining and begins to grow (61). Various factors, including hormones, growth factors, and cytokines regulate the process of embryo implantation, enabling the embryo to connect with the maternal blood supply and receive the nutrients and oxygen required for grow (62). Successful implantation requires the synchronization of blastocysts with implantation ability and the uterus in a receptive state (63). Implantation failure is an important factor leading to pregnancy failure. There are three main reasons for implantation failure: insufficient uterine receptivity, problems with the embryo itself, and systemic causes (63).

In rodents, specific changes occur in the uterus during the pre-implantation period. These changes are divided into three stages: prereceptive, receptive, and nonreceptive stages, corresponding to gestation day (GD) 2&3, GD4, and GD5 in mice. On GD3, the uterus is not yet ready for implantation due to low progesterone(P4) levels. On GD4, with increased progesterone and estrogen secretion, the uterus becomes acceptable and blastocysts can successfully implant. On GD5, the uterus becomes nonreceptive again (64, 65). To examine the spatiotemporal expression of Hmgb1 in uterus during the pre-implantation period, Aikawa S et al. performed *in situ* hybridization using digoxigenin. On GD1-3, Hmgb1 expression is predominantly localized in epithelial cells with some stromal cell localization on GD3. In stroma cells, Hmgb1 signals are primarily observed on GD4&5. Western blotting results show that HMGB1 protein levels decrease with pregnancy progression as assessed on GD4,8&16. The specific spatial and temporal expression of *Hmgb1* at this stage suggests that it plays an essential role in the pre-implantation period (17). Besides, HMGB1, estrogen, and progesterone show regular spatial and temporal expression characteristics during implantation period (17). Progesterone signaling through progesterone receptor (PR) regulates decidualization and endometrial homeostasis by promoting the thickening of the endometrium, inhibiting uterine contraction and regulating the immune system (66–68). Nuclear HMGB1 contributes to successful blastocyst implantation by interregulating with the P4-PR signaling pathway (17, 69). It has been observed that when the *Hmgb1* gene is knockout, the levels of the P4-responsive gene *Hoxa10* in the stromal cells are significantly decreased, thus resulting in reduced efficiency of P4-PR signal transduction (17). This highlights the vital role HMGB1 plays in regulating the P4-PR signaling pathway, which is crucial for successful blastocyst implantation. Additionally, it has been noted that HMGB1 influences the proliferation and differentiation of uterine stromal cells by targeting various proteins such as bone morphogenetic protein (Bmp2), kruppel-like factor 5 (Klf5), cAMP, as well as the G1 phase cell cycle regulator Cyclin D3 (Ccnd3) (16). This emphasizes the multifaceted role nuclear HMGB1 plays in regulating various aspects of blastocyst implantation and signifies its importance in the reproductive process.

Decidualization is the process by which endometrial stromal cells differentiating into decidual stromal cells during early pregnancy, and is accompanied by changes in the immune microenvironment at the maternal-fetal interface. Decidualization plays an extremely important role in embryo implantation, pregnancy establishment and maintenance, and delivery initiation (70). The expression of HMGB1 plays a crucial role in this process. The reduction in HMGB1 expression leads to a cascade of events that inhibits the expression of Bmp2, resulting in the down-regulation of prolactin family 8, subfamily a, member 2 (Prl8a2), an important marker produced by decidual cells. Consequently, uterine stromal cells cannot perform the necessary decidual response, which can ultimately affect further embryonic development (16). In addition to its role in uterine decidualization, HMGB1 also aids in blastocyst implantation by limiting macrophage accumulation and attenuating inflammatory responses in the endometrium. *Hmgb1*-deficient mice have been found to have higher pre-implantation levels of Ccl2 and Csf1, both of which are involved in priming macrophages (17). This ultimately leads to an increase in pre-implantation macrophage accumulation and inflammatory responses. However, it is important to note that the role of HMGB1 in blastocyst implantation is complex, as the elevated expression of HMGB1 in endometrial epithelial cells can decreases the adhesion ability of epithelial cells, thus affecting blastocyst implantation, which is one of the pathogenic mechanisms of recurrent implantation failure (71). Therefore, the precise regulation of HMGB1 expression is critical for successful embryo implantation.

### 3.2 HMGB1 contributes to fetal development: since most of information is related to fetal tissue development

The role of HMGB1 in fetal development is multifaceted and occurs at various stages of gestation. Both endogenous and exogenous HMGB1 affect normal early embryonic development (72). Through its ability to regulate mitochondrial autophagy in the cytoplasm, HMGB1 facilitates cell proliferation and differentiation, thereby aiding in embryo development (73). Interestingly, HMGB1 is found in mouse oocytes and embryos, and its gene expression potentially plays a role in controlling apoptosis during preimplantation via the p53 signaling pathway (72). Bagherpoor et al. found the downregulation of HMGB1 in undifferentiated pluripotent human embryonic stem cells (hESCs) did not affect cell pluripotency. However, in differentiated hESCs, the downregulation of HMGB1 results in decreased telomerase activity, reduced cell proliferation, increased apoptosis, and decreased differentiation to the neural ectodermal lineage (74). Overall, HMGB1 is essential for embryonic development and supports cell growth and differentiation. In addition, HMGB1 protects preimplantation embryonic development by decreasing the expression of Bak and Casp3 genes involved in apoptosis (72, 75).

HMGB1 also promotes the development of the nervous system (19). It has been reported that HMGB1 knockdown in zebrafish embryos leads to forebrain developmental defects in the forebrain due to increased Wnt/β-catenin expression (76). Consistent with these findings, another study showed that HMGB1-dependent CXCL12/CXCR4 signaling pathway is present in the developing mammalian central nervous system (CNS). In addition, a significant reduction in receptor RAGE in the CNS of HMGB1 knockdown mice was accompanied by Wnt/β-catenin overexpression, leading to the downregulation of several developmental factors. These factors include several neurogenesis factors (e.g. Ascl1, Neurod1, Sox2, Tbr2 and Bcl2), developmental factors (e.g. Pax6, Shh, Foxg1 and Emx2) and differentiation factors (BMP2, BMP4 and Tgf1). The decreased expression of these factors leads to increased neuronal apoptosis and decreased proliferation (77). These findings demonstrated that HMGB1 is a key regulator of embryonic brain development.

Moreover, HMGB1 is a crucial factor in the growth and development of bone tissues and limbs. HMGB1 accumulates in the hypertrophic chondrocytes on the growth plate and is then transferred from the cell nucleus to the cytoplasm. During the early stages of cartilage maturation, it is secreted. By acting as a chemotactic factor, it attracts osteoclasts, osteoblasts, and endothelial cells to invade the primary ossification center, thereby promoting intramembranous ossification within the cartilage (78). This promotes the formation of new bone tissue and contributes to the proper development of limbs.

Furthermore, HMGB1 exerts pro-angiogenic effects by inducing MAPK/ERK1/2 activation, cell proliferation, and chemotaxis in endothelial cells from different sources (79, 80). To prove this conclusion, Mitola et al. demonstrated this conclusion by implanting HMGB1-loaded alginate beads on chick chorioallantoic membranes at a developmental stage of 11 days (80). However, the current conclusion lacks evidence in rodent and human embryonic development. Interestingly, the expression of HMGB1 and the receptor RAGE was also observed in dental cell types in the late embryonic stage of rats, indicating that HMGB1 may also promote tooth development (81). In general, current research suggests that HMGB1 plays a crucial role in embryonic development at various stages and in successful pregnancy. However, more research is required to fully understand the complex interactions between HMGB1 and other factors that contribute to successful early life development.

## 4 The role of HMGB1 in female reproductive system diseases

In this section, we will discuss the role of HMGB1 in various conditions that influence female reproductive health by spanning both gynecological disorders and pregnancy-related complications. We aim to present a comprehensive landscape of HMGB1’s role across a timeline that begins prior to conception with issues affecting fertility, such as polycystic ovary syndrome and endometriosis, and extends through varying stages of pregnancy, encapsulating pregnancy complications like unexplained recurrent spontaneous abortion, preeclampsia, gestational diabetes mellitus, and preterm birth. We hope a chronological approach aids in building a comprehensive understanding of the omnipresence of HMGB1’s role throughout the female reproductive journey.

### 4.1 Polycystic ovary syndrome

Polycystic ovary syndrome (PCOS) is a common condition affecting the female reproductive system. It can cause infertility and complications during pregnancy, and affects the physical and mental health of 4%-20% of women of reproductive age globally (82, 83). The main clinical manifestations of PCOS include irregular menstruation (hypomenorrhea or amenorrhea), androgen excess, and multiple ovarian cysts, often accompanied by insulin resistance (IR), obesity, type 2 diabetes, and cardiovascular disease (83, 84). It has also been suggested that inflammation plays an important role in the pathogenesis of PCOS (85). As a molecule associated with several inflammatory diseases, HMGB1 is elevated in both the peripheral blood and follicular fluid of women with PCOS, particularly those with insulin resistance, compared with non-PCOS women (25, 86–90). During the development and maturation of granulosa cells (GC), HMGB1 interacting with TLRs, and may be involved in ovarian innate immunity and ovarian follicle maturation regulated by follicle-stimulating hormone (FSH) (91). However, excess extracellular HMGB1promotes autophagy of granulosa cells (86). Research has shown that inhibition of HMGB1 or the TLR4/NF-κB signaling pathway can improve inflammatory PCOS with insulin resistance (25). Further research is required to understand the mechanisms underlying the relationship between inflammation and PCOS.

### 4.2 Endometriosis

Endometriosis refers to the growth of endometrium-like tissue outside the uterine cavity, such as in the ovaries and the peritoneum (92). Approximately 10% of women of the reproductive age are affected by endometriosis (93). Endometriosis can cause a range of symptoms, including dysmenorrhea, deep pelvic pain, and infertility (94, 95). Although the underlying cause of endometriosis is not fully understood, but inflammation is believed to play a major role in its development (96). Researchers have found that HMGB1 is present in the endometrial cells of women with endometriosis, compared to those who do not (97). HMGB-1 may contribute to the development of endometriosis in part by regulating the inflammatory response and autophagy (97). HMGB1 expression is significantly increased during the secretory phase of the menstrual cycle (31). The extracellular secretion of HMGB1 appears to enhance the proliferation of endometrial stromal cells, contributing to the development of inflammatory responses in the endometrium (98). This effect can be inhibited by TLR4 antagonists and NF-κB inhibitors, suggesting that the HMGB1-TLR4-NF-κB pathway is involved in the development of aseptic inflammation in endometrial tissue (98). These findings were supported by subsequent studies confirming the role of HMGB1 in the pathogenesis of endometriosis (99, 100). The study conducted by Cao et al. not only localized HMGB1 in endometriosis patients but also found that its circulating levels were higher in these patients than in women without the disease, indicating its potential as a biomarker for detecting endometriosis (101). The researchers further examined the relationship between HMGB1 and glycolysis-related indicators pyruvate kinase M2 (PKM2) and hexokinase 2 (HK2). They discovered a positive correlation between their levels with HMGB1 expression, suggesting that HMGB1 may play a role in the pathogenesis of endometriosis by affecting glycolysis (26). In addition, the effects of HMGB1 on endometriosis may be linked to pyroptosis (102). However, it was also observed that HMGB1 was not significantly upregulated in patients with severe endometriosis (103). Therefore, more research is necessary to determine the viability of HMGB1 as a biomarker for endometriosis.

### 4.3 Unexplained recurrent spontaneous abortion

Recurrent spontaneous abortion (RSA) is a common pregnancy complication of two or more failed clinical pregnancies (104). This complication involves multiple factors, such as chromosomal abnormalities, age, antiphospholipid syndrome, uterine malformations, thrombosis, hormonal or metabolic disorders, infections, autoimmunity, sperm quality, and lifestyle issues (105). Despite extensive studies, no apparent causative factor exists for unexplained recurrent spontaneous abortion (URSA) in 50% to 75% of the patients diagnosed with RSA (106). Recent research has indicated that underlying immunological alterations may contribute to the development of URSA. In addition, studies have shown that abnormal expression of HMGB1 may be closely associated with the development of URSA.

Jin et al. found that individuals with HMGB1 rs2249825C/G polymorphism had a higher risk of RSA and also experience higher expression of HMGB1 in the chorionic villi. This indicates a strong association between the two factors (107). Patients with URSA have higher levels of serum HMGB1 compared to normal pregnant women (21, 108, 109), which is also observed in patients with PE. In patients with URSA, the maternal-fetal interface is disorganized with cell arrangement and nuclear rupture issues. There were more infiltrating cells in the chorionic villi and decidua than in normal pregnancies (21, 108, 109). The co-localization of HMGB1 with CD45 and Vimentin, but not CK7, suggests that increased HMGB1 at the maternal-fetal interface is likely due to immune cell secretion and passive release from ecdysteroid stromal cells following necrosis, rather than chorionic epithelial cells (108). Further studies have shown that the immune cells that secrete HMGB1 are predominantly macrophages (21). In both mouse models and human decidua tissues, the receptors RAGE, TLR2, and TLR4 were upregulated in the decidua of the URSA group, along with elevated expression of HMGB1. As a result, the expression levels of the inflammasome NLRP-3 and the pyroptosis-related proteins caspase-1 and GSDMD were also increased. These findings suggest that the role of HMGB1 goes beyond merely amplifying the inflammatory response, and it plays a significant role in the pathogenesis of URSA by inducing pyroptosis. Therefore, HMGB1 amplifies the inflammatory response not only by interacting with RAGE, TLR2, and TLR4 receptors, thereby activating the NF-κB signaling pathway, but also by inducing pyroptosis during the pathogenesis of URSA (21).

Furthermore, the use of HTR8/SVneo cells induced with lipopolysaccharide (LPS) as an *in vitro* model provides an opportunity for researchers to investigate the impact of inflammation on the uteroplacental interface. The experiment conducted by Zhou et al. revealed that the exposure to LPS has a significant impact on the ability of HTR8/SVneo cells to grow and move, which are essential functions for a healthy pregnancy. However, HMGB1 knockdown restores these two abilities and blocks the elevated expression of Beclin1 and LC3 in HTR-8/SVneo cells after LPS induction (110). This suggests that HMGB1 may also be involved in the development of miscarriage through the induction of autophagy. In summary, an unusual increase in HMGB1 expression in the maternal-fetal interface and circulatory system can affectseveral crucial processes in pregnancy, ultimately increasing the likelihood of miscarriage. To decrease the occurrence of URSA and improve the birth rate in women of childbearing age, it is crucial to conduct further studies on the precise mechanism by which HMGB1 mediates URSA.

### 4.4 Preeclampsia

Preeclampsia (PE) is a common characteristic complication of pregnancy, which complicates 2% to 4% of pregnancies globally and causes approximately 46,000 maternal deaths and 500,000 fetal and new-born deaths annually (111). It usually occurs after 20 weeks of gestation and often presents with hypertension, proteinuria, thrombocytopenia, liver and kidney function damage, headaches and other symptoms (111, 112). To date, the etiology and pathogenesis of PE have not been fully elucidated. According to the current research, the main factors involved in the pathogenesis of PE include insufficient recasting the uterine spiral arterioles, disturbance of immune regulation, excessive activation of inflammation, damage of vascular endothelium and genetic factors (111).

Numerous studies have demonstrated that women with PE have elevated levels of HMGB1 in multiple tissues, including plasma, placenta, trophoblast tissue, fetal membrane, and decidua (113–115), particularly those with severe or early-onset PE (116, 117). HMGB1 likely contributes to the pathogenesis of PE through several signaling pathways. For instance, it has been discovered that the levels of HMGB1 are significantly higher in the micro- and nano-vesicles of PE placental explants, which cause endothelial cell activation (118). Additionally, hypoxia-induced high levels of HMGB1 secretion in trophoblast tissue can lead to increased endothelial cell permeability through the TRL4/caveolin-1 (Cav-1) pathway, which may be a key factor in the clinical manifestations of PE hyperalbuminuria and systemic edema (114). Besides, elevated plasma HMGB1 stimulates group 3 innate lymphoid cells (ILC3) differentiation and increases IL-17 production (119). Moreover, in PE patients, the binding of HMGB1 to RAGE enhances the NF-κB signaling pathway, which leads to the elevation of pro-inflammatory cytokines such as IL-6 and TNF-α (120–122). It is known that IL-6 may be involved in the overexpression of placental sFlt-1 (123), and TNF-α can reduce the level of endothelial nitric oxide synthase (124), and the combination of these two causes excessive inflammatory reaction and vascular dysfunction, increased circulating endothelial particles and thrombophilia in the PE mother. Therefore, it can be inferred that HMGB1 is an important mediator for the promotion of PE generation.

Through RAGE-NF-κB-IL-6/CCL2 signaling pathway, HMGB1 has been shown to have a critical role in stimulating adipocytes, leading to the further development of inflammation in pregnant women with PE (125). According to Tangerås et al., the syncytial layer is the most important HMGB1-TLR4 activation site, and elevated HMGB1 in the syncytial layer of patients with PE induces TLR4-dependent IL-8 release through the inflammatory isoform of HMGB1 in placental explants and trophoblasts (126). This plays a significant role in the occurrence of local placental inflammation. In addition, hypoxic trophoblast HMGB1 can also induce human umbilical vein endothelial cells (HUVEC) to produce cytotoxicity and leukocyte arrest, as well as higher expression of cell adhesion molecules (VCAM-1 and ICAM-1), thereby causing cell damage (127). These findings suggest that HMGB1 plays a vital role in the development of PE, and that it is possible to prevent and treating PE through anti-HMGB1 therapy. Further research in this area is necessary to fully understand the role of HMGB1 in PE and its potential as a therapeutic target.

### 4.5 Gestational diabetes mellitus

Gestational diabetes mellitus(GDM) is the most common gestational metabolic disease, which refers to diabetes mellitus with normal glucose metabolism before pregnancy and develops only during pregnancy (128). GDM is a significantly harmful disease to both the mother and fetus. It is associated with a high risk of many adverse pregnancy outcomes, such as pre-eclampsia (PE), infection, fetal growth restriction, giant fetus, miscarriage, and postpartum type 2 diabetes (128, 129). Exploring the pathogenesis and treatment of GDM has always had an important place in obstetrics and gynecological research.

Using a cross-sectional comparison of the plasma levels of HMGB1 in 75 pregnant women with positive glucose tolerance tests and 48 pregnant women with negative glucose tolerance tests, Giacobbe et al. found that circulating levels of HMGB1 were higher in patients with GDM than in women with normal pregnancies (130). However, another study that compared plasma HMGB1 levels in GDM patients and normal pregnant women did not support this finding. No significant correlation were observed between HMGB1 levels and GDM. Surprisingly, another study found that maternal age was significantly associated with HMGB1 in patients with GDM but not in the normal pregnancy group (131). Thus, maternal age, a common risk factor for GDM, may influence the incidence of GDM by interacting with HMGB1. Similarly, Santangelo et al. also discovered no significant difference in the plasma HMGB1 levels between pregnant women with GDM and those with normal glucose tolerance. However, they observed an increase in the expression of HMGB1 protein expression in the fetal membrane tissue of patients with GDM. It is associated with high expression of VPAC2 (a VIP receptor) and RAGE receptors in the omental adipose tissue (22). The interaction between HMGB1 and RAGE has been previously shown to result in the secretion of inflammatory cytokines (132), suggesting that the increased expression of HMGB1 in patients with GDM may contribute to the chronic inflammatory state, which is relevant to obesity and insulin resistance. Further, studies are still needed to explore the relationship between HMGB1 and the pathogenesis of GDM.

### 4.6 Preterm birth

Preterm birth(PTB) is a term used to describe delivery that occurs before the 37th week of gestation (133). The global preterm birth rate is about 10% (134, 135). Approximately 70% of preterm birth are spontaneous, while 30% are related to maternal and/or fetal conditions (136, 137). Preterm premature rupture of membrane (pPROM) refers to the rupture of the amniotic sac (membranes) before 37 weeks’ gestation, which contributes to 30-40% of all preterm birth (137). Studies have shown that intra-amniotic infection and/or inflammation have a significant causal relationship with preterm birth (138, 139). In recent years, several studies have demonstrated elevated levels of HMGB1 in the amniotic fluid and plasma of patients with preterm birth compared to those with normal pregnancies (138, 140–145). HMGB1 induces preterm birth in mouse models (146). These findings suggest that HMGB1 may serve as a new non-invasive biomarker for PTB diagnosis.

In normal pregnancies, HMGB1 levels in the amniotic fluid (AF) are not regulated by gestational age (GA) and are higher at delivery than before delivery (140). HMGB1 promotes immune activation at the fetal-maternal interface, thereby facilitating delivery. Elevated levels of HMGB1 have been observed in the serum of mothers with chorioamnionitis-associated preterm birth (147). Intra-amniotic inflammation induces upregulation of HMGB1 expression and release of HMGB1 in the amnion through inhibition of miR-548 and miR-199a-3p (148, 149). In aseptic inflammation-associated preterm delivery, it was found that HMGB1 was released into the amniotic fluid after undergoing acetylation modification. Additionally, RAGE and TLR2/4 showed a dose-dependent increase in response to HMGB1, ultimately activating the p38MAPK signaling pathway. As a result, the expression of pro-inflammatory cytokines such as IL-1β, IL-6, IL-8, and TNF-α was significantly elevated, promoting inflammation unrelated to infection but somewhat related to aging (142). Previous research has shown that HMGB1 can promote inflammation in the chorioamnion by increasing the mRNA expression and protein concentration of NLRP3 and NOD2 while at the same time mediating the release of mature IL-1β and IL-6 by activating caspase-1 (146). Both IL-1β and IL-6 have been found to play crucial roles in the development of inflammation and can ultimately lead to preterm birth and delivery (150–152).

Furthermore, research has demonstrated that HMGB1 plays a crucial role in promoting the proliferation and activation of iNKT cells during metaphase. Once activated, these iNKT cells act by secrete a range of cytokines and lysis granules, such as IFN-γ, perforin and granzyme B, which may cause damage to both fetal and maternal tissues. As a result, the release of HMGB1 is more enhanced, leading to further inflammation and the promotion of PTB development (153). In addition, one study found that the mRNA expression and protein levels of several molecules, including HMGB1, RAGE, NF-κB/p65, matrix metalloproteinase (MMP)-9, and MMP-2, were significantly increased in the HMGB1-RAGE pathway in pregnant women who had experienced pPROM compared with those who had experienced normal full-term pregnancies. This suggests that the nucleoplasmic translocation of HMGB1 in pPROM placentas may cause it to bind to its receptor RAGE, which then stimulates the activity of NF-κB/p65. The activation of NF-κB/p65 triggers the release of MMP-9 and MMP-2. Therefore, HMGB1 is implicated in the progression of pPROM (154). According to the above researches, a significant relationship exists between HMGB1 and PTB pathogenesis. The prevention and treatment of PTB by targeting anti-HMGB1 has gradually become more encouraging.

The function of HMGB1 in the female reproductive system is twofold. It has been discovered to play a crucial role in the development of various conditions such as preeclampsia, preterm birth, gestational diabetes mellitus, unexplained recurrent spontaneous abortion, polycystic ovary syndrome, and endometriosis. With such a broad range of connections, HMGB1 may act as a biomarker for anticipating the beginning of these ailments. The early detection of HMGB1 levels can help prevent the onset of these health conditions in women through anti-HMGB1 treatment.

## 5 HMGB1-targeted therapeutic agents

### 5.1 Glycyrrhizin

Glycyrrhizin (GL) is a natural compound that is commonly found in large quantities in the roots and rhizomes of Glycyrrhiza glabra (155). It is a triterpene diol conjugate (156). Specifically, it has been discovered that GL forms a direct bond with HMGB1. This occurs when the GL interacts with two shallow concave surfaces that are created by the two arms of the two HMG boxes (157). GL can inhibit HMGB1-TLR4-NF-kB mRNA expression level and has physical interaction with HMGB1 and TLR4 observed in molecular docking (158). This makes it a valuable tool for the treatment of various diseases associated with HMGB1. Initially, GL was used primarily for the treatment of chronic hepatitis. Still, it is becoming increasingly significant in treating various other diseases such as oncology, lung diseases, cardiovascular diseases, sepsis, and more (159–165). Moreover, GL has shown great potential in treating pregnancy-related complications, making them a valuable tool for maternal and fetal health.

In patients with PE, the placenta is defective and trophoblast cells are hypoxic (166), which leads to increased secretion of HMGB1 by hypoxic trophoblastic tissues. The secretion of HMGB1 induces endothelial cell hyperpermeability via the TLR4/caveolin-1 pathway. Consequently, this leads to the development of generalized edema and hyperuria in patients with preeclampsia (114). However, according to Jiang et al., the concentration of 200 mg/ml of GL was found to reduce the permeability of hypoxic JEG-3-CM-induced human umbilical vein endothelial cells (HUVEC) by 89.8% (114). This finding suggests that GL can reduce endothelial cell permeability in patients with PE.

GL has a potent anti-inflammatory effect and can be used to treat pregnancy-related issues. Excessive glucose can cause inflammation in trophoblast cells, leading to increased HMGB1 expression. HMGB1 interacts with TLR4, resulting in the secretion of IL-6 and IL-8, which slows down trophoblast migration. However, GL can reverse the above effect caused by excessive glucose (167). Furthermore, in rodent models of PE, oral GL reduces HMGB1 and inflammatory factors including IL-1 and IL-6, both in serum and in the placenta (121). These results illustrate GL reduces inflammation in pregnancy complications by suppressing HMGB1 release and bioactivity. GL can also block the process of HMGB1-mediated senescence activation through the p38MAPK pathway in fetal membranes and placenta of PTB (142). This is an important finding as it indicates that GL may play a role in preventing preterm birth. Further studies have been conducted on the safety of GL administration during pregnancy. One such study involved the oral gavage of GD10-19 in pregnant rats, which showed no adverse effects on blood pressure and proteinuria (121). This indicates that GL is safe for use during pregnancy, at least in rats. Further research is necessary before recommending its use in other animals or humans.

### 5.2 Recombinant thrombomodulin

Thrombomodulin (TM), also referred to as CD141, is a glycoprotein found on the surface of endothelial cells (168, 169). It has a lectin-like domain that can bind to and neutralize HMGB1 (170–172). Recently, recombinant thrombomodulin (rTM), a commercial form of TM, was found to have anti-inflammatory effects and to improve body function by inhibiting HMGB1 in various diseases (173).

TM is highly expressed in placental trophoblast cells during normal pregnancies (174), and reduced TM expression has been observed in women with placental defects such as PE and miscarriage (175). It has been hypothesized that the TM plays a role in maintaining placental function. Indeed, was discovered that treating a mouse model of recurrent miscarriage induced by angiotensin II (Ang II)-related PE with rTM significantly inhibited multiple pathways mediated by HMGB1. rTM leads to decreased adverse pregnancy outcomes (176).

In PE, effect of elevated HMGB1 binding to its receptors is the activation of the HIF-regulated hypoxic stress response through NF-κB transcriptional upregulation and HIF-1α expression. It also inhibited placental angiogenesis by reducing placental growth factor (PlGF) production and enhancing sFlt-1 expression (120). In mice with pathological pregnancies, the use of rTM hindered the rise of HMGB1 levels in the blood and the production of pro-inflammatory cytokines such as IL-6 and TNF-α in the placenta. Additionally, it decreased the accumulation of HIF-1α protein, increased PlGF expression in the placenta, and reduced the buildup of fibrinogen in the placental vagus region (120). Therefore, rTM ultimately improved the fetal resorption rate and fetal growth restriction in the recurrent miscarriage model and reduced symptoms such as hypertension and proteinuria in the PE model (120, 176, 177). Moreover, rTM is too large to cross the placenta and affect the fetus (177), making it a promising drug for treating pregnancy complications such as pre-eclampsia, recurrent miscarriage, and fetal growth restriction.

### 5.3 Low molecular weight heparin

Low molecular weight heparin (LMWH) is a commonly used anticoagulant for treating pregnancy complications such as recurrent miscarriage, pre-eclampsia, and fetal growth restriction (153, 178). Although there is no evidence for the beneficial effects of heparin in reducing adverse neonatal outcomes (179). This helps to avoid adverse pregnancy outcomes (180, 181). Recent studies have shown that LMWH can bind to HMGB1, resulting in a reduced affinity of HMGB1 for RAGE. This effect may play a role in protecting the placental function and improving pregnancy (182). According to Zenerino et al., after 48 hours of LMWH treatment, the levels of HMGB1, RAGE, IL-6, and TNF-α were found to be reduced in a physiological placental villous explant model (178). This finding suggests that LMWH regulates the HMGB1/RAGE pro-inflammatory axis in human placenta. Moreover, LMWH has been discovered to hinder the growth of iNKT cells when co-cultured with APC in the presence of HMGB1 (178). This finding implies that LMWH may be effective in preventing preterm births without acute chorioamnionitis. In a mouse model of LPS-induced PE, LMWH enhanced pregnancy outcomes by reducing iron-regulator expression and boosting iron absorption (153). Further investigations are required to determine how heparin improves pregnancy rates. This is because heparin binds to both HMGB1 and RAGE (183), making it unclear which molecule is responsible for the inhibition of the HMGB1-RAGE axis. In addition, it is important to be cautious when using LMWH during severe PE as it can stimulate the placental expression and release of sFLT-1 (184).

### 5.4 Other agents

The following drugs can also target HMGB1 to provide new approaches for the treatment of pregnancy-related complications (**Table 1**; **Supplementary Table 1**). Aspirin has potential therapeutic benefits in treating pregnancy complications such as recurrent abortions (189). Salicylic acid (SA) has been found to have binding sites on HMGB1 in a specific domain through nuclear magnetic resonance (NMR) spectroscopy studies (185). Acetylsalicylic acid, commonly known as aspirin, is thought to work by inhibiting HMGB1 through its binding with SA (185). Aspirin has been shown to reduce expression levels of HMGB1 in both decidua tissue and peripheral blood in the mouse model of recurrent miscarriage, as well as decrease expression of receptors RAGE, TLR2 and TLR4 (21, 109). Magnesium sulfate, as a first-line drug for PE treatment, can inhibit vascular endothelial cell (VEC) apoptosis via the miR218-5p/HMGB1 axis (186). MiR-218-5p has the potential to bind to the 3’-UTR of HMGB1 and can negatively regulate the expression of HMGB1 (190). Compared with the rats in the normal group, miR-218-5p expression decreased in the placental tissues and VECs of the rats in the PE, while HMGB1 increased. Magnesium sulfate can reverse the changes and thus play a role in treating PE (186). In addition, studies have shown that Epigallocatechin gallate (EGCG) can reduce the expression of HMGB1 in hypoxic trophoblast cells in a dose-dependent manner. This helps to improve the cells’ angiogenic state and reduce endothelial dysfunction for treating PE (187). A water-soluble derivative of tanshinone IIA called sodium tanshinone IIA sulfonate (STS) has been discovered to inhibit the expression and release of HMGB1 in the hypoxic trophoblast. STS can be absorbed by the intestine and is capable of reversing HMGB1-induced cytotoxicity, leukocyte arrest, as well as the high expression of cell adhesion molecules including VCAM-1 and ICAM-1, which implies STS has the potential to treat PE (127). Researchers have discovered that Clarithromycin can extend gestation in mice with aseptic intra-amniotic inflammation induced by HMGB1. This results in a reduction of preterm birth rates and helps improve neonatal survival (188). Besides, there is a study showing that betamethasone treatment may also prevent preterm birth caused by HMGB1 (139).

**Table 1:** Effects of potential drugs on HMGB1 in the female reproductive system.

Drug	Binding with HMGB1	Effect on diseases in female reproductive system	Refs
Glycyrrhizin	GL can bind to the two shallow concave surfaces created by two arms of two HMG boxes. GL forms hydrogen bonds with amino acid residues of Arg22, Ala38, Ile34, Lys39 and Phe14 og HMGB1.	Reduces HMGB1 and inflammatory factors including IL-1 and IL-6, both in serum and in the placenta of preeclampsia Blocks the process of HMGB1-mediated senescence activation through the p38MAPK pathway in fetal membranes and placenta of preterm birth	(121, 142, 157, 158)
Recombinant thrombomodulin	rTM can bind to HMGB1 with a lectin-like domain and degrade HMGB1.	Hindersd the rise of HMGB1 levels in the blood and the production of pro-inflammatory cytokines such as IL-6 and TNF-α in the placenta of PE Decreases the accumulation of HIF-1α protein, increased PlGF expression in the placenta, and reduced the buildup of fibrinogen in the placental vagus region of PE	(120, 171, 172)
Low molecular weight heparin	LMWH binding with HMGB1 changes the secondary structure of HMGB1 showing the reducing content of β-pleated sheet while increasing content of α-helix.	Reduces the levels of HMGB1, RAGE, IL-6, and TNF-α in placenta Reduces iron-regulator expression and boosts iron absorption in PE	(153, 178, 182)
Aspirin	Aspirin binds to a specific domain oh HMGB1.	Ameliorates the maternal-fetal interface destruction in unexplained recurrent spontaneous abortion by reducing the expression HMGB1 along with its receptors and suppressing pyroptosis activation	(21, 185)
Magnesium sulfate	–	Inhibits vascular endothelial cell apoptosis via the miR218-5p/HMGB1 axis in PE	(186)
Epigallocatechin gallate	–	Improves angiogenic state and reduce endothelial dysfunction by reducing HMGB1 expression in hypoxic trophoblast cells of PE	(187)
Sodium tanshinone IIA sulfonate	–	Reverses HMGB1-induced cytotoxicity, leukocyte arrest, as well as the high expression of cell adhesion molecules including VCAM-1 and ICAM-1 in PE	(127)
Clarithromycin	–	Reduces preterm birth rates and improve neonatal survival in mice with HMGB1-induced aseptic intra-amniotic inflammation	(188)
Betamethasone	–	Restores the normal delivery timing in a PTB model of HMGB1-induced aseptic intra-amniotic inflammation.	(139)

"-" indicates that there are no available studies or information regarding the specific sites where these drugs bind with HMGB1.

## 6 Conclusions and perspectives

**Figure 3:**
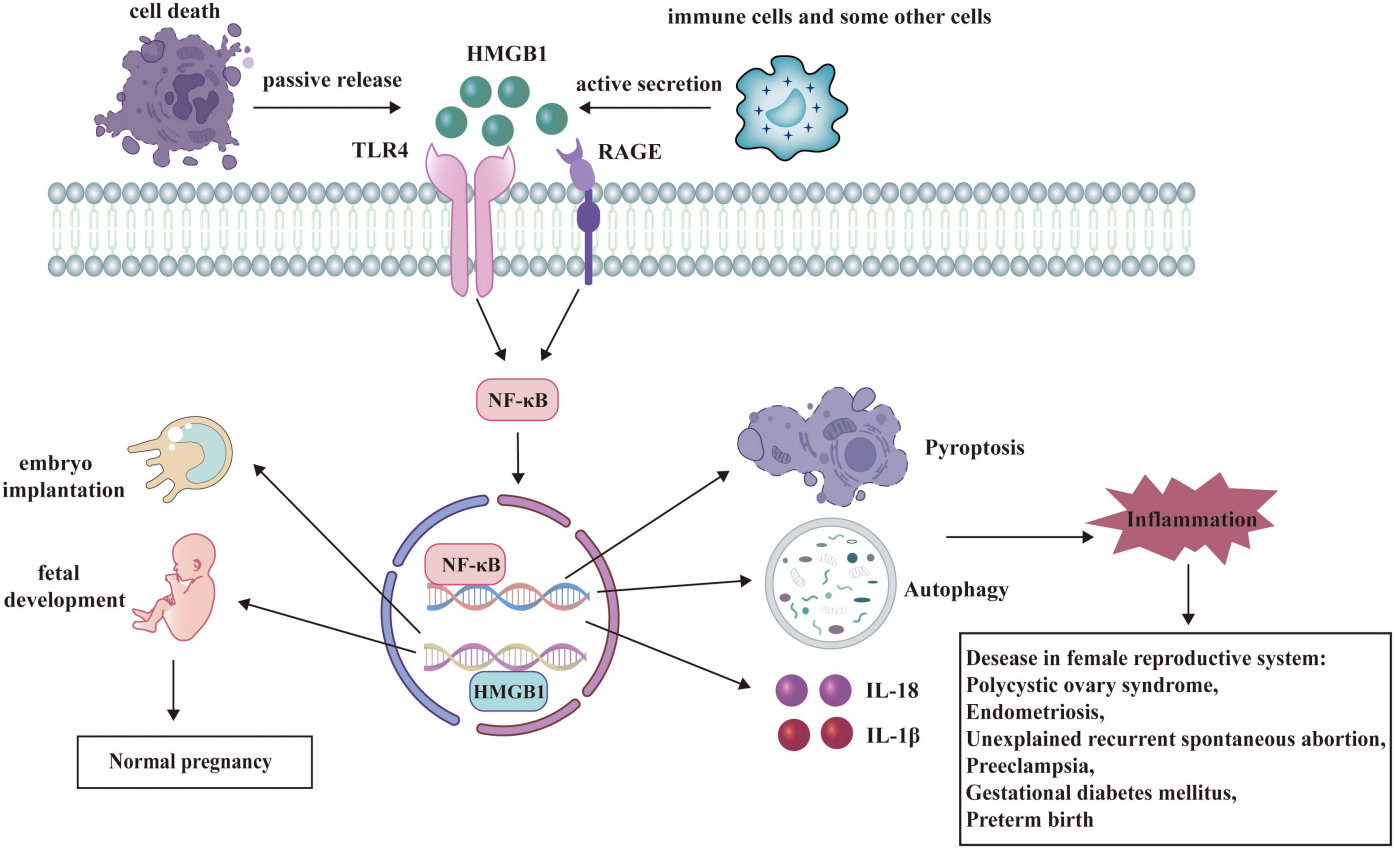
HMGB1 has a dual role in female reproductive system. HMGB1 participates in the regulation of many signaling pathways and plays significant roles in female reproductive system. Under physiological conditions, HMGB1 facilitates embryo implantation and fetal development. As a DAMP, HMGB1 binds to its receptors and activates several pathways such as autophagy, pyroptosis, and release of inflammatory cytokines. These processes contribute to the pathogenesis of diseases in female reproductive system.

This review emphasizes the multiple roles of HMGB1 playing in female reproductive system and some potential HMGB1-targeted therapies (**Figure 3**). Under physiological conditions, HMGB1 is essential for the critical stages of pregnancy, such as uterine decidualization, embryo implantation, and early fetal development. HMGB1 also participates in the pathogenesis of diseases in female reproductive system as a DAMP. Therefore, HMGB1-targeted drugs may offer new ways to prevent or treat these diseases. However, more studies are needed to clarify the following questions (1): The precise mechanism by which HMGB1 plays roles in female reproductive system: Although there has been much research to explore the function of HMGB1, it’s necessary to further figure out every node associated with HMGB1and their connection in female reproductive system. (2) The specific mechanism of HMGB1-targeted therapies in female reproductive system: In this review, we refers to some kinds of promising HMGB1-targeted drugs including glycyrrhizin, recombinant thrombomodulin, low molecular weight heparin, aspirin and so on. But many issues related to them are still unclear. What are the binding targets of these drugs to HMGB1? How do they directly or indirectly regulate the signaling pathway where HMGB1 is located? Will they also target other signaling pathways? What are their administration methods, precautions, and adverse reactions? Is there any new HMGB1 Targeted therapy?

## Author contributions

YR, ZZ, YC and HZ designed the study and edited the final text. YR, ZD, XH and QZ collected the data from publications, developed the database and wrote the manuscript. YR prepared the figures. YR prepared the tables. BC, PZ, ZW, ZZ, YC and HZ contributed to the manuscript revision and critical discussion. All authors contributed to the article and approved the submitted version.
