# Supplementary material for: HMGB1: a double-edged sword and therapeutic target in the female reproductive system
Source: Front Immunol. 2023 Aug 18;14:1238785. doi: 10.3389/fimmu.2023.1238785 (PMC10484633; doi:10.3389/fimmu.2023.1238785)
Supplement: Supplementary file 1 [file DataSheet_1.docx]

Supplementary Material

HMGB1: a double-edged sword and therapeutic target in the female reproductive system

Yu Ren^1, 2, 3^, Zhu Damin^1, 4, 5^, Xingxing Han^1, 4, 5^, Qiqi Zhang^1, 4, 5^,Beili Chen^1, 4, 5^, Ping Zhou^1, 4, 5^, Zhaolian Wei^1, 4, 5^, Zhiguo Zhang^1, 2, 3*^ , Yunxia Cao^1, 2, 3*^ , Huijuan Zou^1, 2, 3*^

^1^Reproductive Medicine Center, Department of Obstetrics and Gynecology, the First Affiliated Hospital of Anhui Medical University, Hefei, China

^2^National Health Commission (NHC) Key Laboratory of Study on Abnormal Gametes and Reproductive Tract (Anhui Medical University), Hefei, China

^3^Key Laboratory of Population Health Across Life Cycle (Anhui Medical University), Ministry of Education of the People’s Republic of China, No 81 Meishan Road, Hefei 230032, Anhui, China

^4^Anhui Province Key Laboratory of Reproductive Health and Genetics, No 81 Meishan Road, Hefei 230032, Anhui, China

^5^Biopreservation and Artificial Organs, Anhui Provincial Engineering Research Center, Anhui Medical University, No 81 Meishan Road, Hefei 230032, Anhui, China

*** Correspondence:**

Huijuan Zou

[hienjoyshine@aliyun.com](mailto:zhj@ahmu.edu.cn;)

Zhiguo Zhang

[zzg_100@163.com](mailto:zhangzhiguo@ahmu.edu.cn;)

Yunxia Cao

caoyunxia6@126.com

# Supplementary Table

**Supplementary Table 1: The study's findings on the impact of HMGB1 and its targeting agents on pregnancy complications.**

| **Pregnancy Complications** | **Trimester of Pregnancy** | **Sample Type** | **Conclusion** | **Blocking** | **Reference** |
| --- | --- | --- | --- | --- | --- |
| PE | Third | Placenta | MgSO_4_ partially prevents vascular endothelial cell (VEC) apoptosis in PE rats via miR218-5p/HMGB1 axis. | MgSO_4_ | (1) |
| PE | Third | Serum and placenta | Excess HMGB1 in PE serum causes increased placental sFlt-1, IL-6, and TNF-α expression, but decreased placental local HMGB1 expression. rTM reduces serum levels of HMGB1, thus providing protection against pregnancy. | rTM | (2) |
| PE | Third | Serum and placenta | Glycyrrhizin significantly reduced elevated HMGB1 and TLR4 expression and inflammatory molecule levels in placenta and serum, and improved pregnancy outcome in preeclamptic rats. | Glycyrrhizin | (3) |
| PE | - | Human umbilical vein endothelial cells (HUVEC) | EGCG attenuated the hypoxia-induced increase in HMGB1 expression in a dose-dependent manner and, through this signaling pathway, attenuated the anti-angiogenic state of hypoxic trophoblast cells, improved cell viability, promoted cell proliferation, and inhibited vascular endothelial dysfunction. | EGCG | (4) |
| PE | Third | Serum and peripheral blood mononuclear cells(PBMC) | Elevated levels of HMGB1 and multiple pro-inflammatory factors in PE serum. | - | (5, 6) |
| PE | Third | Serum | Elevated HMGB1 in PE serum a increases adipocyte expression of IL-6 and CCL2. | - | (7) |
| PE | Third | Placenta | Increased expression of HMGB1 in the placental syncytial layer interacts with TLR4 to increase IL-8 expression and promote aseptic inflammation. | - | (8) |
| PE | Third | Serum | Higher concentrations of HMGB1 and TLR4 in PE serum. | - | (9) |
| PE | Third | Serum and placenta | Increased expression and release of HMGB1 from hypoxic trophoblast cells plays an important role in promoting endothelial particle production, blood coagulation and neutrophil activation, which leads to thrombosis. | - | (10) |
| PE | - | JEG-3 cell line, HUVEC cell line | Hypoxic trophoblast-derived HMGB1 causes increased cytotoxicity and increased VCAM-1 and ICAM-1 expression in human umbilical vein endothelial cells (HUVEC) and leukocyte arrest, and STS can inhibit these effects by targeting HMGB1. | STS | (11) |
| PE | Third | Placenta | Significantly increased levels of HMGB1 in micron and nanovesicles from preeclampsia serum-treated placental explants, resulting in endothelial cell activation. | LMWH | (12) |
| PE | Third | Serum and placenta | Sirtin1 regulates HMGB1 and HSP70-dependent signaling, and the levels of HMGB1 and HSP70 are elevated in the serum of PE mice, but SIRT1 expression is downregulated in the placenta. | - | (13) |
| PE | Third | Serum and placenta | Levels of HMGB1 were increased in both extruded microvesicles and nanovesicles in placental explants treated with preeclamptic serum, which activated endothelial cells and increased cytotoxicity. | - | (14, 15) |
| PE | Third | Placenta | The placentas of women with PE showed significantly increased expression of NLRP3, caspase-1, IL-1β, TNF-α, and HMGB1 compared to those of pregnant women with normal blood pressure. | - | (16) |
| PE | Third | Placenta | HMGB1 expression in syncytial trophoblast cytoplasm is significantly increased in severe PE and early-onset PE compared to normal pregnancy. | - | (17) |
| PE | Third | Serum and placenta | Increased expression of HMGB1 and RAGE in placenta and serum in severe PE compared to normal pregnancy. | - | (18) |
| PE&GDM | Third | Serum | HMGB1 is elevated in the plasma of PE and GDM. |  | (19) |
| PE | Third | Serum | Late pregnancy is associated with elevated HMGB1 levels, both with and without pre-eclampsia. | - | (20) |
| PE | - | Placenta | Active or passive release of HMGB1 derived from hypoxic trophoblasts increases endothelial cell permeability via the TLR4-CAV-1 pathway. | - | (21) |
| PB | Third | Amniotic fluid | Intra-amniotic injection of HMGB1 promotes preterm delivery, an effect that can be reversed by clarithromycin. | Clarithromycin | (22, 23) |
| PB | Third | Amniotic membrane | Senescent amniocyte-derived exosomes package HMGB1 with exosomes and eHMGB1 can lead to RAGE activation iuducing PB in uterine tissue. | - | (24) |
| PB | Third | Decidua | Inappropriate activation of innate immune cells and increased expression of HMGB1 may represent signs of delivery in human pregnancy. | - | (25) |
| PB | Third | Amniotic fluid | Intra-amniotic injection of HMGB1 leads to late preterm delivery and Betamethasone treatment prevents HMGB1-induced preterm delivery. | Betamethasone | (26) |
| PB | Third | Cervical epithelium | miR-199a-3p regulates inflammation through the HMGB1/TLR4/NF-κB pathway. The expression of miR-199a-3p was significantly lower in cervical tissue and the expression levels of HMGB1 and TLR4 were elevated in PB patients compared to full-term pregnancies. | - | (27) |
| PB | Third | Amniotic membrane、amniotic fluid | HMGB1 is released by amniotic epithelial cells and is highly expressed in the amnion of patients with preterm labor and acute chorioamnionitis, with increased secretion of HMGB1 in the amniotic fluid. | - | (28) |
| PB | Third | Chorionic amniotic membrane | HMGB1 concentrations in the chorioamnion are higher in women with spontaneous preterm delivery than in women with full-term spontaneous delivery. HMGB1 induces an inflammatory response in the chorioamnion, which is partially mediated by inflammasome. | - | (29-32) |
| PB | Third | Placenta | HMGB1 secreted by placental senescent cells increases the expression of cellular senescence, sarcoplasmic contraction, and inflammatory genes, thereby promoting human birth through aseptic inflammation induction associated with cellular senescence. | - | (33) |
| PB | Third | Amniotic membrane、amniotic fluid | HMGB1 production by fetal membranes leads to p38MAPK-mediated autocrine activation of senescence-associated inflammation unrelated to infection. | - | (34) |
| GDM | - | Sw.71（Human early pregnancy trophoblast cell line） | Hyperglycemia triggers sterile inflammation of the trophoblast via HMGB1-activated TLR4, an effect that can be reversed by glycyrrhizin. | Glycyrrhizin | (35) |
| GDM | Third | Serum | Plasma HMGB1/IFN-γ is increased in pathological pregnancies thereby stimulating ILC3 differentiation resulting in increased IL-17 production and therefore producing inflammation. | - | (19) |
| GDM | Third | Serum | There is no difference in HMGB1 serum levels in GDM compared to pregnant women with NGT (normal glucose tolerance). | - | (36) |
| GDM | Third | Serum | There was no significant correlation between HMGB1 and GDM. | - | (37) |
| GDM | Third | Serum | Elevated HMGB1 levels in late pregnancy are associated with gestational diabetes and insulin resistance. | - | (38) |
| URSA | First | Serum and decidua | HMGB1 was increased in both circulating levels and decidua in patients with URSA. The high expression of HMGB1 at the maternal-fetal interface in URSA patients is actively secreted by infiltrating immune cells, while decidual stromal cells may also passively release HMGB1 during necrosis. And HMGB1 may exacerbate aseptic inflammation at the maternal-fetal immune interface through pyroptosis. | Aspirin | (39-41) |
| URSA | - | HTR8/SVneo cells | HMGB1 is involved in LPS-induced trophoblastic inflammation via autophagy. | - | (42) |

**Reference**

1. Zheng J, Tian M, Liu L, Jia X, Sun M, Lai Y. Magnesium Sulfate Reduces Vascular Endothelial Cell Apoptosis in Rats with Preeclampsia Via the Mir-218-5p/Hmgb1 Pathway. *Clinical and Experimental Hypertension (New York, NY : 1993)* (2022) 44(2):159-66. doi: 10.1080/10641963.2021.2013492.

2. Oda H, Nagamatsu T, Schust DJ, Cabral H, Miyazaki T, Iriyama T, et al. Recombinant Thrombomodulin Attenuates Preeclamptic Symptoms by Inhibiting High-Mobility Group Box 1 in Mice. *Endocrinology* (2021) 162(4). Epub 2021/01/07. doi: 10.1210/endocr/bqaa248.

3. Liu F, Yang X, Xing J, Han K, Sun Y. Glycyrrhizin Potentially Suppresses the Inflammatory Response in Preeclampsia Rat Model. *Pregnancy Hypertens* (2021) 23:34-40. Epub 2020/11/15. doi: 10.1016/j.preghy.2020.10.007.

4. Zhong M, Peng J, Xiang L, Yang X, Wang X, Zhu Y. Epigallocatechin Gallate (Egcg) Improves Anti-Angiogenic State, Cell Viability, and Hypoxia-Induced Endothelial Dysfunction by Downregulating High Mobility Group Box 1 (Hmgb1) in Preeclampsia. *Med Sci Monit* (2020) 26:e926924. Epub 2020/10/16. doi: 10.12659/msm.926924.

5. Romão-Veiga M, Bannwart-Castro CF, Borges VTM, Golim MA, Peraçoli JC, Peraçoli MTS. Increased Tlr4 Pathway Activation and Cytokine Imbalance Led to Lipopolysaccharide Tolerance in Monocytes from Preeclamptic Women. *Pregnancy Hypertens* (2020) 21:159-65. Epub 2020/06/15. doi: 10.1016/j.preghy.2020.06.002.

6. Jabalie G, Ahmadi M, Koushaeian L, Eghbal-Fard S, Mehdizadeh A, Kamrani A, et al. Metabolic Syndrome Mediates Proinflammatory Responses of Inflammatory Cells in Preeclampsia. *Am J Reprod Immunol* (2019) 81(3):e13086. Epub 2019/01/08. doi: 10.1111/aji.13086.

7. Akasaka J, Naruse K, Sado T, Uchiyama T, Makino M, Yamauchi A, et al. Involvement of Receptor for Advanced Glycation Endproducts in Hypertensive Disorders of Pregnancy. *International Journal of Molecular Sciences* (2019) 20(21). doi: 10.3390/ijms20215462.

8. Tangerås LH, Silva GB, Stødle GS, Gierman LM, Skei B, Collett K, et al. Placental Inflammation by Hmgb1 Activation of Tlr4 at the Syncytium. *Placenta* (2018) 72-73:53-61. Epub 2018/12/07. doi: 10.1016/j.placenta.2018.10.011.

9. Li J, Huang L, Wang S, Zhang Z. Increased Serum Levels of High Mobility Group Protein B1 and Calprotectin in Pre-Eclampsia. *Int J Gynaecol Obstet* (2018) 142(1):37-41. Epub 2018/03/24. doi: 10.1002/ijgo.12491.

10. Hu Y, Yan R, Zhang C, Zhou Z, Liu M, Wang C, et al. High-Mobility Group Box 1 from Hypoxic Trophoblasts Promotes Endothelial Microparticle Production and Thrombophilia in Preeclampsia. *Arterioscler Thromb Vasc Biol* (2018) 38(6):1381-91. Epub 2018/04/14. doi: 10.1161/atvbaha.118.310940.

11. Zhao M, Feng Y, Xiao J, Liang J, Yin Y, Chen D. Sodium Tanshinone Iia Sulfonate Prevents Hypoxic Trophoblast-Induced Endothelial Cell Dysfunction Via Targeting Hmgb1 Release. *J Biochem Mol Toxicol* (2017) 31(7). Epub 2017/03/16. doi: 10.1002/jbt.21903.

12. Zenerino C, Nuzzo AM, Giuffrida D, Biolcati M, Zicari A, Todros T, et al. The Hmgb1/Rage Pro-Inflammatory Axis in the Human Placenta: Modulating Effect of Low Molecular Weight Heparin. *Molecules* (2017) 22(11). Epub 2017/11/18. doi: 10.3390/molecules22111997.

13. Yin Y, Feng Y, Zhao H, Zhao Z, Yua H, Xu J, et al. Sirt1 Inhibits Releases of Hmgb1 and Hsp70 from Human Umbilical Vein Endothelial Cells Caused by Il-6 and the Serum from a Preeclampsia Patient and Protects the Cells from Death. *Biomed Pharmacother* (2017) 88:449-58. Epub 2017/01/26. doi: 10.1016/j.biopha.2017.01.087.

14. Xiao X, Xiao F, Zhao M, Tong M, Wise MR, Stone PR, et al. Treating Normal Early Gestation Placentae with Preeclamptic Sera Produces Extracellular Micro and Nano Vesicles That Activate Endothelial Cells. *J Reprod Immunol* (2017) 120:34-41. Epub 2017/04/26. doi: 10.1016/j.jri.2017.04.004.

15. Shao J, Zhao M, Tong M, Wei J, Wise MR, Stone P, et al. Increased Levels of Hmgb1 in Trophoblastic Debris May Contribute to Preeclampsia. *Reproduction* (2016) 152(6):775-84. Epub 2016/11/01. doi: 10.1530/rep-16-0083.

16. Weel IC, Romao-Veiga M, Matias ML, Fioratti EG, Peracoli JC, Borges VT, et al. Increased Expression of Nlrp3 Inflammasome in Placentas from Pregnant Women with Severe Preeclampsia. *Journal of Reproductive Immunology* (2017) 123:40-7. doi: 10.1016/j.jri.2017.09.002.

17. Chen Q, Yin YX, Wei J, Tong M, Shen F, Zhao M, et al. Increased Expression of High Mobility Group Box 1 (Hmgb1) in the Cytoplasm of Placental Syncytiotrophoblast from Preeclamptic Placentae. *Cytokine* (2016) 85:30-6. Epub 2016/06/11. doi: 10.1016/j.cyto.2016.06.001.

18. Zhu L, Zhang Z, Zhang L, Shi Y, Qi J, Chang A, et al. Hmgb1-Rage Signaling Pathway in Severe Preeclampsia. *Placenta* (2015) 36(10):1148-52. Epub 2015/08/26. doi: 10.1016/j.placenta.2015.08.006.

19. Barnie PA, Lin X, Liu Y, Xu H, Su Z. Il-17 Producing Innate Lymphoid Cells 3 (Ilc3) but Not Th17 Cells Might Be the Potential Danger Factor for Preeclampsia and Other Pregnancy Associated Diseases. *Int J Clin Exp Pathol* (2015) 8(9):11100-7. Epub 2015/12/01.

20. Pradervand PA, Clerc S, Frantz J, Rotaru C, Bardy D, Waeber B, et al. High Mobility Group Box 1 Protein (Hmgb-1): A Pathogenic Role in Preeclampsia? *Placenta* (2014) 35(9):784-6. Epub 2014/07/22. doi: 10.1016/j.placenta.2014.06.370.

21. Jiang R, Cai J, Zhu Z, Chen D, Wang J, Wang Q, et al. Hypoxic Trophoblast Hmgb1 Induces Endothelial Cell Hyperpermeability Via the Trl-4/Caveolin-1 Pathway. *J Immunol* (2014) 193(10):5000-12. Epub 2014/10/24. doi: 10.4049/jimmunol.1303445.

22. Galaz J, Romero R, Arenas-Hernandez M, Farias-Jofre M, Motomura K, Liu Z, et al. Clarithromycin Prevents Preterm Birth and Neonatal Mortality by Dampening Alarmin-Induced Maternal-Fetal Inflammation in Mice. *BMC Pregnancy Childbirth* (2022) 22(1):503. Epub 2022/06/21. doi: 10.1186/s12884-022-04764-2.

23. Gomez-Lopez N, Romero R, Plazyo O, Panaitescu B, Furcron AE, Miller D, et al. Intra-Amniotic Administration of Hmgb1 Induces Spontaneous Preterm Labor and Birth. *American Journal of Reproductive Immunology* (2016) 75(1):3-7. doi: 10.1111/aji.12443.

24. Radnaa E, Richardson LS, Sheller-Miller S, Baljinnyam T, de Castro Silva M, Kumar Kammala A, et al. Extracellular Vesicle Mediated Feto-Maternal Hmgb1 Signaling Induces Preterm Birth. *Lab Chip* (2021) 21(10):1956-73. Epub 2021/05/20. doi: 10.1039/d0lc01323d.

25. Kato M, Negishi Y, Shima Y, Kuwabara Y, Morita R, Takeshita T. Inappropriate Activation of Invariant Natural Killer T Cells and Antigen-Presenting Cells with the Elevation of Hmgb1 in Preterm Births without Acute Chorioamnionitis. *Am J Reprod Immunol* (2021) 85(1):e13330. Epub 2020/08/28. doi: 10.1111/aji.13330.

26. Galaz J, Romero R, Arenas-Hernandez M, Panaitescu B, Para R, Gomez-Lopez N. Betamethasone as a Potential Treatment for Preterm Birth Associated with Sterile Intra-Amniotic Inflammation: A Murine Study. *Journal of Perinatal Medicine* (2021) 49(7):897-906. doi: 10.1515/jpm-2021-0049.

27. Peng J, Jiang J, Wang H, Feng X, Dong X. Mir‑199a‑3p Suppresses Cervical Epithelial Cell Inflammation by Inhibiting the Hmgb1/Tlr4/Nf‑Κb Pathway in Preterm Birth. *Mol Med Rep* (2020) 22(2):926-38. Epub 2020/05/30. doi: 10.3892/mmr.2020.11184.

28. Son GH, Kim Y, Lee JJ, Lee KY, Ham H, Song JE, et al. Microrna-548 Regulates High Mobility Group Box 1 Expression in Patients with Preterm Birth and Chorioamnionitis. *Sci Rep* (2019) 9(1):19746. Epub 2019/12/26. doi: 10.1038/s41598-019-56327-9.

29. Plazyo O, Romero R, Unkel R, Balancio A, Mial TN, Xu Y, et al. Hmgb1 Induces an Inflammatory Response in the Chorioamniotic Membranes That Is Partially Mediated by the Inflammasome. *Biology of Reproduction* (2016) 95(6):130. doi: 10.1095/biolreprod.116.144139.

30. Romero R, Miranda J, Chaiworapongsa T, Korzeniewski SJ, Chaemsaithong P, Gotsch F, et al. Prevalence and Clinical Significance of Sterile Intra-Amniotic Inflammation in Patients with Preterm Labor and Intact Membranes. *American Journal of Reproductive Immunology (New York, NY : 1989)* (2014) 72(5):458-74. doi: 10.1111/aji.12296.

31. Romero R, Chaiworapongsa T, Savasan ZA, Hussein Y, Dong Z, Pedro Kusanovic J, et al. Clinical Chorioamnionitis Is Characterized by Changes in the Expression of the Alarmin Hmgb1 and One of Its Receptors, Srage. *Journal of Maternal-Fetal & Neonatal Medicine* (2012) 25(6):558-67. doi: 10.3109/14767058.2011.599083.

32. Sheller-Miller S, Urrabaz-Garza R, Saade G, Menon R. Damage-Associated Molecular Pattern Markers Hmgb1 and Cell-Free Fetal Telomere Fragments in Oxidative-Stressed Amnion Epithelial Cell-Derived Exosomes. *Journal of Reproductive Immunology* (2017) 123. doi: 10.1016/j.jri.2017.08.003.

33. Menon R, Behnia F, Polettini J, Saade GR, Campisi J, Velarde M. Placental Membrane Aging and Hmgb1 Signaling Associated with Human Parturition. *Aging* (2016) 8(2):216-30.

34. Bredeson S, Papaconstantinou J, Deford JH, Kechichian T, Syed TA, Saade GR, et al. Hmgb1 Promotes a P38mapk Associated Non-Infectious Inflammatory Response Pathway in Human Fetal Membranes. *PLoS One* (2014) 9(12):e113799. Epub 2014/12/04. doi: 10.1371/journal.pone.0113799.

35. Heim KR, Mulla MJ, Potter JA, Han CS, Guller S, Abrahams VM. Excess Glucose Induce Trophoblast Inflammation and Limit Cell Migration through Hmgb1 Activation of Toll-Like Receptor 4. *Am J Reprod Immunol* (2018) 80(5):e13044. Epub 2018/09/04. doi: 10.1111/aji.13044.

36. Santangelo C, Filardi T, Perrone G, Mariani M, Mari E, Scazzocchio B, et al. Cross-Talk between Fetal Membranes and Visceral Adipose Tissue Involves Hmgb1-Rage and Vip-Vpac2 Pathways in Human Gestational Diabetes Mellitus. *Acta Diabetol* (2019) 56(6):681-9. Epub 2019/03/02. doi: 10.1007/s00592-019-01304-x.

37. Hill AV, Menon R, Perez-Patron M, Carrillo G, Xu X, Taylor BD. High-Mobility Group Box 1 at the Time of Parturition in Women with Gestational Diabetes Mellitus. *Am J Reprod Immunol* (2019) 82(5):e13175. Epub 2019/07/30. doi: 10.1111/aji.13175.

38. Giacobbe A, Granese R, Grasso R, Salpietro V, Corrado F, Giorgianni G, et al. Association between Maternal Serum High Mobility Group Box 1 Levels and Pregnancy Complicated by Gestational Diabetes Mellitus. *Nutr Metab Cardiovasc Dis* (2016) 26(5):414-8. Epub 2016/04/20. doi: 10.1016/j.numecd.2016.02.007.

39. Zhu D, Zou H, Liu J, Wang J, Ma C, Yin J, et al. Inhibition of Hmgb1 Ameliorates the Maternal-Fetal Interface Destruction in Unexplained Recurrent Spontaneous Abortion by Suppressing Pyroptosis Activation. *Front Immunol* (2021) 12:782792. Epub 2022/01/11. doi: 10.3389/fimmu.2021.782792.

40. Wang J, Zhu D, Yin J, Ma C, Peng X, Zou H, et al. Upregulated Hmgb1 Levels in Maternal-Fetal Interface of Patients with Unexplained Recurrent Spontaneous Abortion from Different Sources. *J Matern Fetal Neonatal Med* (2021):1-8. Epub 2021/05/05. doi: 10.1080/14767058.2021.1918084.

41. Zou H, Yin J, Zhang Z, Xiang H, Wang J, Zhu D, et al. Destruction in Maternal-Fetal Interface of Ursa Patients Via the Increase of the Hmgb1-Rage/Tlr2/Tlr4-Nf-Kappab Signaling Pathway. *Life Sci* (2020) 250:117543. Epub 2020/03/15. doi: 10.1016/j.lfs.2020.117543.

42. Zhou F, Wang Y, Tan Y, Wu C, Chen Y. Hmgb1 Regulates Lipopolysaccharide-Induced Cellular Dysfunction in Htr8/Svneo Cells: Implications for the Role of Hmgb1 in Unexplained Spontaneous Miscarriage. *Placenta* (2021) 112:16-22. Epub 2021/07/10. doi: 10.1016/j.placenta.2021.06.012.
